# Enhancement of Antioxidant and Antibacterial Activities of *Salvia miltiorrhiza* Roots Fermented with *Aspergillus oryzae*

**DOI:** 10.3390/foods9010034

**Published:** 2020-01-01

**Authors:** Keumok Moon, Jaeho Cha

**Affiliations:** 1 Department of Microbiology, Pusan National University, Busan 46241, Korea; moonko81@nate.com; 2 Microbiological Resource Research Institute, Pusan National University, Busan 46241, Korea

**Keywords:** *Salvia miltiorrhiza*, *Aspergillus oryzae*, fermentation, antibacterial activity

## Abstract

The roots of *Salvia miltiorrhiza* are known to exhibit antioxidant and antibacterial activities. To improve the antioxidant and antibacterial activities of *S. miltiorrhiza* roots, the roots were fermented with *Aspergillus oryzae* at 25 °C for 3 weeks. The non-fermented (SME) and fermented (SMBE) roots of *S. miltiorrhiza* were extracted with 70% ethanol, respectively, and then fractionated with organic solvents. By fermentation, total phenolic and flavonoid contents, as well as antioxidant activity of SMBE, were increased by about 1.2 to 1.3 times compared with those of SME. The antibacterial activity of SMBE was also twice as high as that of SME. The antibacterial activity of SMBE against *Bacillus cereus* was lower in the *n*-hexane and chloroform fractions, but higher in the ethyl acetate and *n*-butanol fractions, compared with those of SME. These results indicate that the bioactive components of *S. miltiorrhiza* roots exhibiting antibacterial activity were converted to more polar compounds by fermentation of *A. oryzae*. Gas chromatography and mass spectrometry (GC-MS) and LC-MS analyses of SME and SMBE demonstrate that these changes are due to the acylation of dihydrofuran-2(*3H*)-one, dealkylation of 4-methylbenzene-1,2-diol and 4-ethylbenzene-1,2-diol, and esterification of hexadecanoic acid and (9Z, 12Z)-octadec-9,12-dienoic acid during fermentation.

## 1. Introduction

*Salvia miltiorrhiza,* also known as red sage, is a perennial plant of the Labiatae family. It has been used clinically to treat and prevent numerous diseases, such as cardiovascular disease, hyperlipidemia, and cerebrovascular disease, worldwide. It has been used as a tea or ingredients for foods and medicine. More than 70 compounds have been isolated and structurally identified from *S. miltiorrhiza*. The main components of *S. miltiorrhiza* can be divided into two groups: hydrophilic compounds, including salvianolic acids, and lipophilic compounds, including tanshinones [1]. It was previously reported that ethanol extracts of *S. miltiorrhiza* exhibited antibacterial activity against several oral pathogenic microbes and antifungal activity against *Candida albicans* [2,3]. The diterpenoid tanshinones and phenolic acids from the cultured hairy roots of *S. miltiorrhiza* exhibited antimicrobial activities against both gram-positive and gram-negative bacteria, as well as fungi [4]. The essential oil extracted from the roots of *S. miltiorrhiza*, the main component of which was an ethyl hexadecanoate, exhibited antibacterial and antioxidant activities [5]. A strong correlation between the phenolic acids of *S. miltiorrhiza* and in vitro antioxidant activity has also been reported. Seventeen components, including 12 phenolic compounds and 5 tanshinones, were analyzed from 50 batches of *S. miltiorrhiza,* and it was found that phenolic compounds contributed more to antioxidant activity than tanshinones [6]. Given its high value as various pharmacological activities, *S. miltiorrhiza* is widely cultivated in many places for industrial purposes. Approximately 80 million kilograms of *S. miltiorrhiza* are required for the food and pharmaceutical industry each year [7,8]. However, the yield of these bioactive compounds varies depending on culture conditions [9].

Microbial fermentation processes have been used for the purpose of releasing high amounts of phenolic compounds and fatty acids from plant materials, as well as to convert phenolic compounds into various derivatives that can exert better biological activities [10,11,12]. Generally, bacteria, such as *Bacillus* and lactic acid bacteria, yeasts, such as *Saccharomyces* and *Cryptococcus*, and fungi, such as *Aspergillus* and *Rhizopus*, are used in these processes. *Aspergillus oryzae* has been used in many fermented foods to release biologically active compounds from plants and to promote their biological activities, because various enzymes are strongly secreted from this fungus. Capsaicin is converted to various new metabolites by the enzymatic reactions of *A. oryzae*, such as hydroxylation, isomerization, and oxidation [13]. It has also been reported that fermentation of rice bran by *A. oryzae* and *Rhizopus oryzae* increases the antioxidant activity and inhibitory activity of tyrosinase and elastase, suggesting that fermented rice bran extracts could be used as ingredients in cosmetics, as well as in antioxidants [14]. Additionally, soybean isoflavones could be transformed to hydroxyisoflavones with strong antioxidant activity during fermentation by *A. oryzae* [15]. The coconut oil extracted from coconut cream, treated with various starters, including *Lactobacillus bulgaricus* and *A. oryzae,* showed that the fermented coconut oil was composed of 64% medium chain fatty acids with strong antibacterial activity [12].

In this study, we assessed the effect of fermentation by *A. oryzae* on the antioxidant and antibacterial activities of ethanol extracts and further organic solvent fractions of *S. miltiorrhiza* roots. The antioxidant activity, as well as the total phenolic and flavonoid contents of the ethanol extracts of the fermented (SMBE) roots of *S. miltiorrhiza* were increased, compared with those of non-fermented (SME) roots of *S. miltiorrhiza*. In addition, the antibacterial activity of SMBE against the gram-positive bacteria was also increased compared with that of SME. Gas chromatography and mass spectrometry (GC-MS) analyses of SME and SMBE demonstrated that the increase of these activities is due to the various enzymatic reactions of *A. oryzae*, such as acylation, dealkylation, and esterification.

## 2. Materials and Methods

### 2.1. Materials

The roots of *S. miltiorrhiza* were purchased in the local market in Seoul, Korea, in June 2017. All solvents for extraction were of Low Particulate grade and purchased from SK Chemicals (Ulsan, Korea). Glucose, gallic acid, quercetin dihydrate, the Folin–Ciocalteu reagent, aluminum chloride, anthrone, sulfuric acid, dimethyl sulfoxide (DMSO), 2,2-diphenyl-1-hydrazyl (DPPH), and 2,4,6-tris(2-pyridyl)-1,3,5-triazine (TPTZ) were purchased from Sigma–Aldrich (St. Louis, MO, USA). Sodium carbonate, potassium acetate, ascorbic acid, and iron (III) chloride hexahydrate were purchased from Junsei Chemical Co., Ltd. (Tokyo, Japan). Methanol was purchased from Burdick & Jackson (Ulsan, Korea). Malt extract broth and brain heart infusion (BHI) were purchased from Difco (Sparks, MD, USA), and tryptic soy broth (TSB) was purchased from MB cell (Seoul, Korea).

### 2.2. Microorganisms and Growth Conditions

*A. oryzae* KCTC 6983 was purchased from the Korean Collection for Type Cultures (KCTC) in the Korea Research Institute of Bioscience and Biotechnology (Daejeon, Korea) and grown at 25 °C on a malt extract agar (MEA) plate. For the antibacterial activity assay, *Bacillus cereus* was grown in luria bertani (LB) broth. *Staphylococcus aureus* ATCC25923, *Listeria monocytogenes* ATCC15313, *Pseudomonas aeruginosa* ATCC15442, and *Klebsiella pneumoniae* ATCC4352 were grown in TSB. *Streptococcus iniae* ATCC29178 and *Streptococcus parauberis* were grown in BHI broth. *Vibrio fluvialis* KCTC 2473 and *Vibrio mimicus* KCTC 2737 were grown in TSB, containing 1% sodium chloride. All tests were performed at 37 °C for 24 h.

### 2.3. Sample Preparation of S. miltiorrhiza Ethanol Extracts

The roots of *S. miltiorrhiza* were ground using a commercial blender, and 50 g of the ground roots were weighed in a flask, which was then sterilized at 121 °C for 20 min and cooled at room temperature. Extraction was conducted with 70% ethanol (EtOH) by sonication for 60 min, and the supernatant was collected. This process was repeated three times, and the supernatant was mixed and filtered (No. 2, Toyo Roshi Kaisha, Ltd., Japan). The supernatant was concentrated with a rotary vacuum evaporator at 60 °C and lyophilized. The dried ethanol extract (SME, 17.2 g) powder was resolved in 10% ethanol and subsequently fractionated with *n*-hexane, chloroform, ethyl acetate (EtOAc), *n*-butanol (*n*-BuOH), and water (Figure S1). Each fraction was lyophilized and dissolved in DMSO at a concentration of 50 mg/mL for the measurement of antibacterial activity.

### 2.4. Solid-State Fermentation

Solid-state fermentation was performed by the method described by Razak et al. [14]. After sterilization of the roots in the same way as non-fermented roots, 50 mL of distilled water was added to the roots. Next, 1% of *A. oryzae* spores (OD_620 nm_ = 1) was added to the flask, mixed well with a sterilized spatula, and incubated at 25 °C for 21 days. The sample was extracted and fractionated by the same method as the sample preparation of SME. The weight of the dried ethanol extract (SMBE) powder was 7.1 g. To rule out the effects of *A. oryzae*, the mycelium was cultured in a MEA plate. The mycelia were incubated at 25 °C for 1 week, harvested, and extracted in the same method as non-fermented roots.

### 2.5. Determination of Total Carbohydrate Content

Total carbohydrate content (TCC) was evaluated using the method described by Leyva et al. [16]. The anthrone reagent was dissolved in concentrated sulfuric acid (98%) at a concentration of 0.1% before analysis. SME and SMBE were dissolved in DMSO at a concentration of 200 μg/mL. A 100 μL of sample was mixed with 200 μL of anthrone solution and kept at 4 °C for 10 min. The mixture was then incubated at 100 °C for 20 min and cooled at room temperature for 20 min. A total of 200 μL of each mixture was transferred to a microplate, and the absorbance at 620 nm was determined. Samples were measured in triplicate. Glucose was used as a standard, and TCC was expressed as gram of glucose equivalent per gram.

### 2.6. Determination of Phenolic and Flavonoid Contents

Phenolic content was determined according to the Folin-Ciocalteu method described by Zhang et al. [17]. SME and SMBE were dissolved in DMSO at various concentrations ranging from 0.2 to 5 mg/mL. Twenty microliters of sample was mixed with 100 μL of 10% (*v*/*v*) Folin–Ciocalteu reagent. After standing for 5 min, 80 μL of 7.5% (*w*/*v*) sodium carbonate (Na_2_CO_3_) was added and mixed. The mixture was incubated for 30 min in the dark at room temperature, and then the absorbance was measured at 765 nm. Gallic acid was used as a standard, and the phenolic content was expressed as mg of gallic acid equivalent (GAE) per gram. Flavonoid content was measured by the slightly modified method described by Chang et al. [18]. A total of 20 μL of the sample were mixed with 100 μL of methanol, 20 μL of 2% (*w*/*v*) aluminum chloride, 20 μL of 0.2 M potassium acetate, and 80 μL of distilled water. The absorbance of the reaction mixture was measured at 415 nm. Quercetin was used as a standard, and the flavonoid content was expressed as mg of quercetin equivalent (QE) per gram.

### 2.7. Antioxidant Assay

The ability of a sample to scavenge DPPH free radical was determined by the method described by Kim et al. with minor modification [19]. Briefly, 10 μL of the sample at various concentrations, ranging from 0.02 to 0.5 mg/mL, was mixed with 90 μL of 0.1 M Tris-HCl (pH 7.5) and 200 μL of 0.1 mM DPPH, diluted in methanol in a 96-well plate, and allowed to stand at room temperature for 30 min. The absorbance was measured at 517 nm and compared with a quercetin standard curve. The DPPH value was expressed as μg of quercetin per gram. The antioxidant capacity of the sample was measured by ferric reducing antioxidant power (FRAP) assay, based on the method described by Benzie et al. with some modification [20]. The FRAP reagent was freshly prepared before analysis. A total of 300 mM acetate buffer (pH 3.6), 10 mM TPTZ in 40 mM HCl, and 20 mM FeCl_3_·6H2O were mixed at a ratio of 10:1:1. The reaction was started by adding 200 μL of the FRAP reagent to 100 μL of the sample at various concentrations, ranging from 0.1 to 1.0 mg/mL, and the mixture was incubated for 30 min at 37 °C in a dark room. The absorbance was detected at 593 nm and compared with a FeSO_4_ standard curve. The FRAP value was expressed as mg of FeSO_4_ per gram.

### 2.8. Antibacterial Activity Assay

The minimum inhibitory concentration (MIC) of *S. miltiorrhiza* extracts was determined by the method described by Xiang et al. [21]. Briefly, the extract was prepared by serial twofold dilution in each medium. Bacteria were added at 5 × 10^6^ CFU/mL to each sterile medium. The assay plates were incubated at 37 °C for 24 h, and the growth inhibition assay was monitored by measuring the absorbance at 600 nm. For the disk agar diffusion assay, *B. cereus* was used as an indicator strain. The bacteria were adjusted to 5 × 10^7^ CFU/mL, and 200 μL of the bacteria was mixed with 5 mL of top agar, and this mixture was poured onto an LB agar plate. Sterile paper disks (8 mm diameter, Toyo Roshi Kaisha Ltd., Tokyo, Japan) were placed on the plates, and 20 μL of the samples were loaded onto the disks. Plates were incubated at 37 °C for 24 h, and diameters of the inhibition zones around the disks were measured. The stability of the *S. miltiorrhiza* extracts was tested by disk assay, and the samples were stored according to the method described by Kozikowski et al. with modification [22]. Briefly, the samples were dissolved in DMSO at a concentration of 50 mg/mL and each sample was incubated at 37 °C. To test the stability against freeze-thaw, each sample was subjected to a freezing and thawing cycle. One cycle was defined as a freezing condition at −20° C for 22 h and a thawing condition at 37 °C for 2 h. This cycle was repeated 25 times, and the samples were collected every fifth cycle. To test thermal stability, the samples were incubated at 100 °C and 121 °C for 15 min.

### 2.9. Liquid Chromatography and Mass Spectrometry Analysis

The phenolic compounds and tanshinones of *S. miltiorrhiza* extracts were analyzed by an ultra- performance liquid chromatography and quadrupole-time-of-flight mass spectrometry (UPLC-Q-TOF MS) system. The ethanol extracts (2 μL) of SME and SMBE were injected into Waters ACQUITY I-Class UPLC system (Waters, Milford, MA, USA). An ACQUITY UPLC BEH C_18_ column (2.1 mm × 100 mm, 1.7 μm; Waters) was applied for the analyses, and the UPLC condition for the component analysis of *S. miltiorrhiza* extracts was performed, according to the method described by Zeng et al. [23]. The Q-TOF mass spectrometer (maXis HD^TM^, Bruker, Germany) was operated in both positive and negative modes with a capillary voltage of 4500 V and an end plate offset of 500 V. The scanning mass range (*m*/*z*) was from 50 to 1000. Nitrogen was used as the nebulizing gas at a flow rate of 8 L/min at a temperature of 200 °C, and at a pressure of 0.8 bar. A Bruker Compass Data Analysis 4.2 (Bruker, Billerica, MA, USA) was used to coordinate the LC-MS system.

### 2.10. Gas Chromatography and Mass Spectrometry Analysis

The component analysis of *S. miltiorrhiza* extracts was conducted using a gas chromatography and mass spectrometry (GC-MS) system (GCMS QP-2010Ultra, Shimadzu, Japan), using a DB-5 MS capillary column (30 m × 0.25 mm × 0.25 μm; Agilent). Helium was used as the carrier gas at a flow rate of 1 mL/min. Chromatographic conditions were as follows: The extract (1 μL) was injected in split mode with a ratio of 1/20 at 280 °C. The initial temperature of the oven was maintained at 60 °C for 2 min, then increased to 200 °C at a rate of 10 °C/min, and finally raised to 320 °C at a rate of 5 °C/min and held for 20 min. The mass analyzer was set to scan from 40 to 600 atomic mass units. Peak identification was carried out by comparison of the experimental mass spectrum in the National Institute of Standards and Technology (NIST14) and Wiley 9 GC-MS libraries.

### 2.11. Statistical Analysis

All experiments were performed in triplicate. Data were analyzed using SigmaPlot software (version 12.5; Systat, San Jose, CA, USA) and expressed as the mean ± standard deviation. A two-tailed *t*-test was used to determine significant differences between variables, and *p* < 0.05 was considered to indicate a statistically significant difference.

## 3. Results

### 3.1. Extraction Yield and TCC of SME and SMBE

Extraction yields were calculated on the basis of the weight of dried roots of *S. miltiorrhiza*. The yield of SME and SMBE extracted with 70% ethanol from 50 g of roots of *S. miltiorrhiza* was 34.4% (17.2 g) and 14.2% (7.1 g), respectively. Further fractions with increasing polarity, *n*-hexane, chloroform, EtOAc, n-BuOH, and water fraction were carried out (Table 1). After fermentation by *A. oryzae*, the extraction yield by ethanol was significantly decreased. The major yield difference between the SME and SMBE was due to the water fraction. The yield of *n*-hexane, chloroform, and water fraction of SMBE was lower than that of SME, whereas the yield of EtOAc fraction was not changed, and the *n*-BuOH fraction was slightly increased after fermentation. The TCC of SME and SMBE were 12.9 and 1.7 g. The decrease in TCC in the SMBE was presumed to be due to the consumption of sugars, which were used as carbon sources by *A. oryzae* during fermentation. The effect on antioxidant and antibacterial activity of *A. oryzae* was also measured because the mycelium was not removed from the fermented roots. The fungus was incubated in the MEA media and harvested. The weight of the mycelium was 4.79 g and the yield of the mycelium extracted with 70% ethanol (AoryE) was 7.3% (0.35 g).

### 3.2. TPC and TFC of SME and SMBE

Phenolic compounds and flavonoids are considered high-level antioxidants owing to their abilities to scavenge free radicals, but they cannot be extracted easily from plant cells as they mainly exist as insoluble bound form conjugates with cell wall components through ester, ether, or glycosidic bonds [11]. To increase the release of phenolic compounds from the roots of *S. miltiorrhiza*, the roots were fermented by *A. oryzae*. The phenolic content and flavonoid content of SME and SMBE were 71.3 ± 0.8 mg GAE and 219.9 ± 12.3 mg GAE/g and 4.3 ± 0.1 mg QE and 12.9 ± 0.5 mg QE/g, respectively. The phenolic and flavonoid content of the AoryE were 21.1 ± 2.8 mg GAE/g and 3.4 ± 0.2 mg QE/g, respectively. The total phenolic content (TPC) and total flavonoid content (TFC) of SME were 1.23 g and 74.5 mg, respectively, and the TPC and TFC of SMBE were 1.56 g and 91.5 mg, respectively. The TPC and TFC of SMBE were increased by 1.3- and 1.2-fold, respectively, compared with those of SME. Although the yield of the phenolic content per weight of dried roots of *S. miltiorrhiza* was the highest in the EtOAc fraction, the TPC of SME and SMBE were the highest in the water fraction, followed by the n-BuOH and EtOAc fractions (Table 1). The TPC of the water fraction of SMBE especially increased by 1.4-fold, compared with that of SME, which seemed to contribute to the increase in the TPC of SMBE.

The yield of the flavonoid content per weight of dried roots of *S. miltiorrhiza* was the highest in the *n*-hexane and the chloroform fraction (Table 1). However, the TFC of SME and SMBE were the highest in the *n*-hexane and the water fraction, respectively. In particular, the TFC yield of SMBE was decreased by about 0.63- and 0.53-fold in the *n*-hexane and chloroform fractions, but increased by about 1.85- and 1.23-fold in the EtOAc and the water fractions, respectively, compared with that of SME. These results indicate that phenolic and flavonoid compounds in *S. miltiorrhiza* roots were converted to slightly polar substances by fermentation.

### 3.3. Antioxidant Activity of SME and SMBE

Several assays, including 2,2azinobis (3-ethyl-benzothiazoline-6-sulfonic acid) (ABTS), DPPH, oxygen radical absorption capacity (ORAC) for radical scavenging capacity, and FRAP for ferric reducing potential, have been frequently used to estimate antioxidant capacities in fresh fruit, vegetables, and foods. Among them, DPPH and FRAP assays are highly reproducible test methods [24]. The abilities to scavenge radicals of SME and SMBE were examined. The DPPH radical scavenging activities of SME, SMBE, and AoryE were 81.1, 281.8, and 6.3 μg QE/g, respectively. The increase in DPPH radical scavenging activity in SMBE is presumably due to the increase in the phenolic content of SMBE. Further fractionation of SME and SMBE showed that DPPH activity was the highest in the EtOAc and *n*-BuOH fractions, but the antioxidant activities between SME and SMBE were not significantly different in the two fractions (Table 2). DPPH radical scavenging activities of SMBE were decreased in the *n*-hexane, EtOAc, and *n*-BuOH fractions, respectively, compared with those of SME. However, the activities of the chloroform and water fractions of SMBE were increased compared to those of SME. The FRAP activities of SME, SMBE, and AoryE were 1.5, 4.9, and 0.2 mg FeSO_4_/g, respectively. The FRAP activity measurement of SMBE and SME showed the same trend as the DPPH method. As expected, the antioxidant activity of SME and SMBE in each fraction was proportional to the TPC content of SME and SMBE. AoryE showed phenolic content of 21.1 mg GAE/g and flavonoid content of 3.4 mg QE/g, but the AoryE exhibited much lower antioxidant activity than that of SME or SMBE.

### 3.4. Antibacterial Activity and Stability of SME and SMBE

Powdered fractions of organic solvent extraction were dissolved in DMSO for the assessment of antibacterial activity. Both SME and SMBE showed antibacterial activities only against gram-positive bacteria, not gram-negative bacteria (Table 3). AoryE did not show antibacterial activity against both gram-positive and gram-negative bacteria. SME showed a high level of antibacterial activity against *B. cereus* and *S. aureus*, as well as a low level of antibacterial activity against *L. monocytogenes*. Antibacterial activities based on the MIC results were increased after fermentation by *A. oryzae*. MIC values against *B. cereus*, *S. iniae*, *S. parauberis* and *L. monocytogenes* were reduced twofold. *S. aureus* showed the same MIC, but *S. aureus* grew more slowly in SMBE than SME at a concentration of 512 μg/mL. Strong inhibitory activity of SME and SMBE against *B. cereus* was observed in the *n*-hexane and chloroform fractions (Table 4). Interestingly, the antibacterial activities wdecreased in the *n*-hexane and chloroform fractions but increased in the EtOAc and *n*-BuOH fractions after fermentation. This result means that bioactive components of *S. miltiorrhiza* roots that possess antibacterial activity have been converted to more polar compounds through a fermentation process.

**Figure 1:**
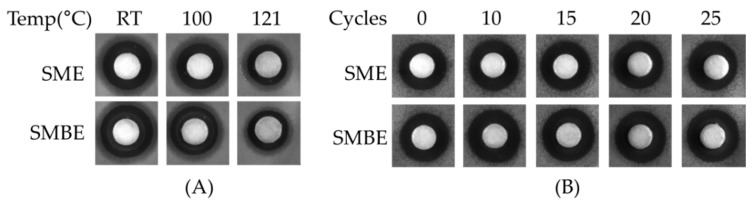
(**A**) Antibacterial activity against *B. cereus* by heat treatment of *S. miltiorrhiza* ethanol extracts. Each sample was incubated at 100 °C or 121 °C for 15 min. (**B**) Antibacterial activity against *B. cereus* in accordance with freeze-thaw cycles of *S. miltiorrhiza* ethanol extracts. Each sample was repeated for 25 cycles of freezing and thawing.

The stability of SME and SMBE was tested against the indicator strain *B. cereus* using disk diffusion assay. The antibacterial activities of SME and SMBE were not significantly affected by heat and freeze-thawing, as shown in Figure 1. Both samples were stable at 100 °C but were slightly affected when the temperature rose to 121 °C. They were stable during the 25 cycles of freezing and thawing. The stabilities of SME and SMBE stored at 37 °C were measured by antibacterial activities against *B. cereus*. The antibacterial activities of SME and SMBE were maintained for up to 1 week and 2 weeks, respectively, and then started to decrease. The antibacterial activities of SME and SMBE were observed for up to 3 and 5 months, respectively.

### 3.5. Identification and Analysis of Metabolites of SME and SMBE

**Figure 2:**
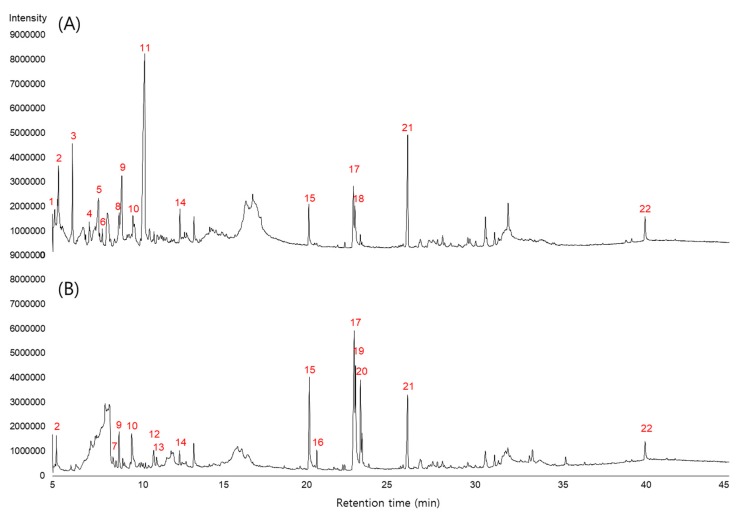
Gas chromatography and mass spectrometry (GC-MS) chromatogram of (**A**) SME and (**B**) SMBE.

LC-MS and GC-MS analyses were performed to identify the compounds of SME and SMBE and to determine how the bioactive components changed after *A. oryzae* fermentation. In GC-MS analysis, the compounds with 85% or more similarity with the library were identified. A total of 27 components, including phenolic compounds, tanshinones, furans and pyrans, and fatty acids were detected and identified in SME and SMBE (Figure 2 and Figure S2, Table 5 and Table 6). Among them, 12 compounds were associated with antioxidant activity and 18 compounds with antibacterial activity. (Table 5 and Table 6). Most of the furans and pyrans except for 5-acetyldihydrofuran-2*(3H)*-one and 4-hydroxy-4-methyltetrahydro-2H-pyran-2-one were decreased or disappeared, whereas the fatty acids, except for (9Z,12Z,15Z)-octadeca-9,12,15-trienoic acid, were increased after fermentation. A total of six fatty acids were detected in SME or SMBE, of which two fatty acids were increased by about threefold, and three new fatty acids were produced after fermentation. GC-MS analysis of AoryE revealed six compounds, including hexadecanoic acid, hexadecanoic acid, and ethyl esters (Figure S3 and Table S1). However, these compounds were detected at levels lower than 1/20, compared to SMBE.

Phenolic acids are the main components of antioxidant activity. Rosmarinic acid and salvianolic acid B, the major antioxidant components of *S. miltiorrhiza*, were detected in SME but not in SMBE. After fermentation, nine unidentified compounds converted from these two phenolic acids were newly generated. Benzene-1,2-diol was increased and 4-methylbenzene-1,2-diol was newly produced, but 4-ethylbenzene-1,2-diol was decreased (Table 5). Tanshinones are the major antibacterial components of *S. miltiorrhiza*. Among the tanshinone family, dihydrotanshinone I, which is known to be a major antibacterial compound of *S. miltiorrhiza*, was reduced, but the cryptotanshinone and tanshinone IIA were increased after fermentation (Table 6). To summarize these results, the fatty acids, phenolic compounds, and tanshinones that were involved in the antioxidant and antimicrobial activity were increased after fermentation, and their overall increase seemed to affect the increase in the antioxidant and antibacterial activities of SMBE.

## 4. Discussion

The purpose of this study was to investigate whether the antioxidant and antibacterial activities of *S. miltiorrhiza* roots can be enhanced through microbial fermentation and how the substances affecting the activity are converted by it. We measured the changes of the TPC and TFC, as well as the antioxidant and antibacterial activities of the ethanol extracts of *S. miltiorrhiza* roots. The compounds affecting the antioxidant and/or antibacterial activities of SME and SMBE were also identified by GC-MS and LC-MS analyses. Furthermore, the enzymatic reactions that are thought to be involved in substance conversion were predicted.

The TPC and TFC of ethanol extracts from *S. miltiorrhiza* roots were increased by 1.3- and 1.2-fold, respectively, after fermentation. Most of the increases in the TPC and TFC were found in the water fractions, indicating that the phenolic and flavonoid compounds in *S. miltiorrhiza* roots were transferred to slightly polar substances by fermentation. Wen et al. reported that the TPC of the *S. miltiorrhiza* water extract was increased by about 6.2-fold by fermentation with *A. oryzae* NCH 42 [36]. Xing et al. also showed that the TPC of fermented *S. miltiorrhiza* roots with *Geomyces luteus* was enhanced by about 1.91-fold, compared to that of non-fermented roots; especially, salvianolic acid B was increased by about twofold [37]. The antioxidant activities of SMBE were also higher than those of SME. The increase of the antioxidant activity of *S. miltiorrhiza* roots by fermentation with *A. oryzae* was proportional to the increase of the TPC. Previously, it has been reported that the total antioxidant capacities of *S. miltiorrhiza* water extracts were enhanced by about 1.5- to 2.1-fold after fermentation by *A. oryzae* NH42, compared with those of the non-fermented water extracts [36]. It has also been reported that the DPPH and FRAP activities of fermented samples of rice bran were significantly increased, compared with those of the non-fermented rice bran [14].

In our study, LC-MS and GC-MS analyses showed that 12 compounds were associated with antioxidant activity. Although salvianolic acid B and rosmarinic acid, which are known to be the main antioxidant components of *S. miltiorrhiza* disappeared, nine derivative compounds were newly generated by fermentation with *A. oryzae*. It is thought that the increase in the antioxidant activity of SMBE compared with SME is due to the overall increase of the TPC and new phenolic derivatives identified by LC-MS and GC-MS analyses. The effects on antioxidant and antimicrobial activities of *A. oryzae* itself were also evaluated because *A. oryzae* could not be removed from fermented roots. AoryE exhibited lower phenolic and flavonoid content than SME or SMBE and displayed much lower antioxidant activity and almost no antibacterial activity. Therefore, the effect of *A. oryzae* was negligible.

SME and SMBE showed antimicrobial activity only against gram-positive bacteria, and the MIC of SMBE was two times lower than that of SME, except that of *S. aureus*. Wen et al. reported that the antibacterial activities of *S. miltiorrhiza, Trichosanthes kirilowii,* and *Glycyrrhizae radix* water extracts were increased slightly after fermentation against various pathogens, including *B. cereus*, *S. aureus*, and *Salmonella enterica* [36]. Of the identified compounds, 18 compounds were associated with antibacterial activities. Zhao et al. reported that phenolic acids (rosmarinic acid and caffeic acid) and tanshinones (dihydrotanshinone I, tanshinone I, tanshinone IIA, and cryptotanshinone) extracted from the hairy roots of *S. miltiorrhiza* showed antimicrobial activity [4]. In our study, although dihydrotanshinone I was decreased, cryptotanshinone and tanshinone IIA were increased after fermentation (Table 6). All six fatty acids found in SME are known to exhibit antibacterial activity. Five out of the six fatty acids were increased after fermentation. In particular, the increase of unsaturated fatty acids (peaks 17, 19, 20) was much higher than that of saturated fatty acids (peaks 15, 16). Solid-state fermentation of rice bran using *Rhizopus oryzae* resulted in a 20% decrease in saturated fatty acids, but a 5% increase in unsaturated fatty acid [38].

**Figure 3:**
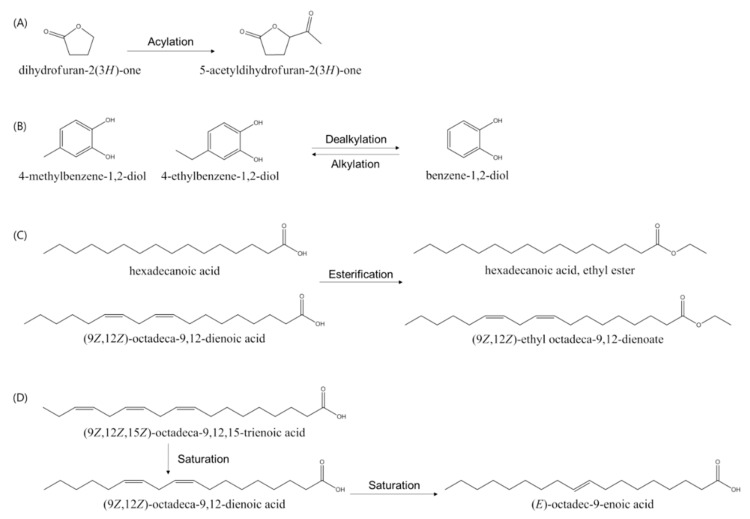
Proposed reaction of bioactive compounds of *S. miltiorrhiza* ethanol extracts during fermentation. (**A**) acylation; (**B**) dealkylation and alkylation; (**C**) esterification; (**D**) saturation.

Further fractionation of SME and SMBE indicated that the antibacterial activity was decreased in the *n*-hexane and chloroform fractions; however, it was increased in the EtOAc and *n*-BuOH fractions. This shift of compounds showing the antibacterial activity may be due to changes in the polarity of bioactive compounds. The GC-MS results revealed that the bioactive compounds with antioxidant and/or antibacterial activities were converted to more polar compounds by various enzymatic reactions during the fermentation of *S. miltiorrhiza*. It was speculated that the following reactions occurred during the fermentation process (Figure 3). Acylation that can convert dihydrofuran-2(3H)-one (peak 1) to 5-acetyldihydrofuran-2(3H)-one (peak 7) may contribute to increased water solubility or biological activities of the compound. Li et al. suggested an efficient method for acylation of gastrodin to improve the low bioavailability of gastrodin, which has therapeutic effects on central nervous system diseases. The acylation of gastrodin was performed by the *A. oryzae* whole-cell catalyst, and the conversion rate was the highest when tetrahydrofuran was used as an organic solvent [39]. Khmelnitsky et al. synthesized water-soluble paclitaxel derivatives through enzymatic acylation of paclitaxel, a potent antimitotic agent, the most soluble of which were 100–1000 times more soluble than paclitaxel [40]. Guo et al. synthesized acylated dihydromyricetin, which is a more potent antioxidant than dihydromyricetin and more soluble in the peanut oil phase [41]. Dealkylation, such as the conversion of 4-ethylbenzene-1,2-diol (peak 14) to benzene-1,2-diol (peak 10), may also affect water solubility. The water solubility of benzene-1,2-diol is 40 times higher than that of 4-ethylbenzene-1,2-diol. Alkylation, such as the conversion of benzene-1,2-diol to 4-methylbenzene-1,2-diol or 4-ethylbenzene-1,2-diol, may contribute to the enhancement of antibacterial and antioxidant activities. Li et al. reported that 4-methylbenzene-1,2-diol or 4-ethylbenzene-1,2-diol increased antiviral activity, compared with benzene-1,2-diol [42]. Esterification, such as the conversion of hexadecanoic acid (peak 15) to ethyl hexadecanoic acid (peak 16), may affect solubility and antimicrobial activity. Sedgwick et al. measured the solubility of saturated fatty acid esters and saturated fatty acids in various solvents [43,44]. The solubility of saturated fatty acid esters was 5 to 50 times higher than that of saturated fatty acids in various solvents. Some fatty acids and their esters exhibited strong antimicrobial activity against several human pathogens [29,30,31]. Therefore, the saturation may also affect the antibacterial activities of the compounds. Huang et al. showed that the antibacterial activity of unsaturated fatty acids varies among oral pathogens [31].

In conclusion, solid state fermentation of *S. miltiorrhiza* roots by *A. oryzae* increased TPC and thereby antioxidant and antibacterial activity. Organic solvent fractionation of bioactive components exhibiting antibacterial activity of *S. miltiorrhiza* roots revealed that they were converted to more polar properties by fermentation of *A. oryzae*. The identification of bioactive compounds by GC-MS and LC-MS analyses demonstrates that these changes of property of compounds were due to acylation, dealkylation, and esterification by fermentation.

## Figures and Tables

**Table 1:** Extraction yield and phenolic and flavonoid content of each solvent fraction of non-fermented (SME) and fermented (SMBE) roots of *S. miltiorrhiza*.

Sample	Extraction Yield (g)		Phenolic Content (mg GAE/g)		Flavonoid Content (mg QE/g)	
	SME	SMBE	SME	SMBE	SME	SMBE
*n*-Hexane	0.97	0.41	33.6 ± 1.3 (32.6) ^1^	30.1 ± 2.6 *** (12.3)	21.6 ± 0.2 (21.0)	32.3 ± 0.0 *** (13.2 ***)
Chloroform	0.59	0.22	41.2 ± 2.5 (24.3)	103.2 ± 3.9 *** (22.7)	28.3 ± 0.5 (16.7)	40.6 ± 0.6 *** (8.9 ***)
EtOAc	0.62	0.62	543.5 ± 19.0 (337.0)	623.0 ± 12.6 ** (386.3 **)	13.3 ± 0.4 (8.2)	24.3 ± 0.9 *** (15.1 ***)
*n*-BuOH	1.25	1.67	426.8 ± 17.3 (533.5)	342.3 ± 2.7 *** (571.6 *)	7.9 ± 0.5 (9.9)	5.2 ± 0.9 * (8.7)
Water	13.79	4.21	42.2 ± 2.8 (581.9)	186.0 ± 2.5 *** (783.1 ***)	1.3 ± 0.2 (17.9)	5.3 ± 0.3 *** (22.3)

Data are expressed as mean ± standard deviation (*n* = 3). ^1^ Parentheses indicate the total phenolic content (TPC) or total flavonoid content (TFC) × extraction yield; * *p* < 0.05, ** *p* < 0.01, *** *p* < 0.001.

**Table 2:** Antioxidant activities of each solvent fraction of SME and SMBE.

Sample	DPPH (μg QE/g)		FRAP (mg FeSO_4_/g)	
	SME	SMBE	SME	SMBE
*n*-Hexane	24.5 ± 0.3 (20.9) ^1^	17.6 ± 0.5 *** (7.2 ***)	0.7 ± 0.1 (0.7)	0.6 ± 0.1 * (0.2 **)
Chloroform	43.1 ± 0.2 (25.4)	98.5 ± 4.1 (21.7)	0.8 ± 0.1 (0.5)	1.8 ± 0.0 * (0.4 *)
EtOAc	750.9 ± 8.3 (465.6)	699.2 ± 19.9 ** (433.5 **)	14.9 ± 0.7 (9.2)	13.4 ± 0.8 ** (8.3 **)
*n*-BuOH	392.7 ± 5.4 (490.9)	347.3 ± 25.7 ** (580.0 **)	8.2 ± 0.5 (10.3)	6.4 ± 0.1 ** (10.7)
Water	39.7 ± 1.3 (547.5)	160.1 ± 16.3 (674.0 *)	0.9 ± 0.1 (12.4)	3.1 ± 0.1 *** (13.1)

Data are expressed as mean ± standard deviation (*n* = 3). ^1^ Parentheses indicate the total 2,2-diphenyl-1-hydrazyl (DPPH) or ferric reducing antioxidant power (FRAP) activity (DPPH or FRAP × extraction yield); * *p* < 0.05, ** *p* < 0.01, *** *p* < 0.001.

**Table 3:** Minimum inhibitory concentration (MIC) values of SME and SMBE against various pathogens.

Bacteria		SME (μg/mL)	SMBE (μg/mL)
Gram (+)	*Bacillus cereus*	128	64
	*Staphylococcus aureus*	256	256
	*Listeria monocytogenes*	2048	1024
	*Streptococcus iniae*	512	256
	*Streptococcus parauberis*	512	256
Gram (-)	*Pseudomonas aeruginosa*	ND ^1^	ND
	*Klebsiella pneumoniae*	ND	ND
	*Escherichia coli*	ND	ND
	*Vibrio fluvialis*	ND	ND
	*Vibrio mimicus*	ND	ND

^1^ Not detected (ND).

**Table 4:** MIC value of each solvent of SME and SMBE against *B. cereus.*

Solvent Fraction	SME (μg/mL)	SMBE (μg/mL)
EtOH	128	64
*n*-Hexane	<2	8
Chloroform	4	16
EtOAc	1024	256
*n*-BuOH	ND ^1^	4096
Water	ND	ND

^1^ Not detected (ND).

**Table 5:** Compounds detected by GC-MS analysis of *S. miltiorrhiza* ethanol extracts.

Peak No.	RT (min)	Area		Name of Compound	Activity ^2^	Ref
		SME	SMBE			
1	5.1	2,021,039	ND ^1^	dihydrofuran-2(*3H*)-one		
2	5.3	6,839,225	2,364,683	2-hydroxycyclopent-2-enone		
3	6.2	11,684,632	ND	2,4-dihydroxy-2,5-dimethylfuran-3(*2H*)-one		
4	7.2	2,621,991	ND	3-nitrobut-1-ene		
5	7.7	10,327,938	ND	4-hydroxy-2,5-dimethylfuran-3(*2H*)-one	2	[25]
6	7.9	1,108,052	ND	5-hydroxy-6-methyl-2H-pyran-4(*3H*)-one		
7	8.6	ND	1,085,995	5-acetyldihydrofuran-2(*3H*)-one		
8	8.9	5,064,272	ND	3-acetyl-3-hydroxydihydrofuran-2(*3H*)-one		
9	9.1	15,999,437	4,860,418	3,5-dihydroxy-6-methyl-2H-pyran-4(*3H*)-one	2	[26]
10	9.7	3,175,165	6,158,327	benzene-1,2-diol	1,2	[27]
11	10.4	63,567,813	ND	5-(hydroxymethyl)furan-2-carbaldehyde		
12	10.9	ND	3,084,106	4-hydroxy-4-methyltetrahydro-2H-pyran-2-one	2	[28]
13	11.1	ND	1,184,908	4-methylbenzene-1,2-diol	1,2	[27]
14	12.5	3,903,321	1,754,609	4-ethylbenzene-1,2-diol	1	
15	20.1	5,873,955	15,800,575	hexadecanoic acid	1,2	[29]
16	20.6	ND	1,634,484	hexadecanoic acid, ethyl ester	1,2	[30]
17	22.7	10,155,786	29,314,886	(9Z,12Z)-octadeca-9,12-dienoic acid	2	[31]
18	22.8	8,594,663	ND	(9Z,12Z,15Z)-octadeca-9,12,15-trienoic acid	2	[32]
19	22.9	ND	16,887,502	octadec-9-enoic acid	2	[31]
20	23.2	ND	10,363,933	(9Z,12Z)-ethyl octadeca-9,12-dienoate	2	[31]
21	26.0	18,898,107	12,189,786	ferruginol	1,2	[33]
22	40.0	4,118,012	3,229,685	γ-sitosterol	1,2	[34]

^1^ Not detected (ND); ^2^ 1: antioxidant; 2: antibacterial.

**Table 6:** Compounds detected by LC-MS analysis of *S. miltiorrhiza* ethanol extracts.

Peak No.	R.T. (min)	Ion Mode	Area		Name of the Compound	Activity ^2^	Ref
			SME	SMBE			
1	6.0	ES-	1,434,150	ND ^1^	rosmarinic acid	1,2	[4]
2	6.3	ES-	6,285,563	ND	salvianolic acid B	1,2	[35]
3	9.3	ES+	10,401,970	1,136,992	dihydrotanshinone I	1,2	[4]
4	9.8	ES+	57,574,444	71,574,320	cryptotanshinone	1,2	[4]
5	10.3	ES+	65,527,940	95,715,976	tanshinone IIA	1,2	[4]

^1^ Not detected (ND); ^2^ 1: antioxidant; 2: antibacterial.

## Supplementary Materials

The following are available online at https://www.mdpi.com/2304-8158/9/1/34/s1. Figure S1: Sequential fractionation of EtOH extract with organic solvents. Figure S2: LC-MS chromatogram of SME (A and C) and SMBE (B and D). Figure S3: GC-MS chromatogram of AoryE. Table S1: Compounds detected by GC-MS analysis of AoryE.

## References

[1] Wang B.Q. (2010). *Salvia miltiorrhiza*: Chemical and pharmacological review of a medicinal plant. J. Med. Plants Res..

[2] Deng J., Zhang D., Yang W. (2006). An in vitro experiment on the antimicrobial effects of ethanol extract from *Salvia miltiorrhiza* Bunge on several oral pathogenic microbes. Shanghai Kou Qiang Yi Xue.

[3] Lee H.S., Kim Y. (2016). Antifungal activity of *Salvia miltiorrhiza* against *Candida albicans* is associated with the alteration of membrane permeability and (1, 3)-β-d-glucan synthase activity. J. Microbiol. Biotechnol..

[4] Zhao J., Lou J., Mou Y., Li P., Wu J., Zhou L. (2011). Diterpenoid tanshinones and phenolic acids from cultured hairy roots of *Salvia miltiorrhiza* Bunge and their antimicrobial activities. Molecules.

[5] Lou J., Mao Z., Shan T., Wang Q., Zhou L. (2014). Chemical composition, antibacterial and antioxidant properties of the essential oils from the roots and cultures of *Salvia miltiorrhiza*. J. Essent. Oil Bear. Plants.

[6] Zhang X., Yu Y., Cen Y., Yang D., Qi Z., Hou Z., Han S., Cai Z., Liu K. (2018). Bivariate correlation analysis of the chemometric profiles of Chinese wild *Salvia miltiorrhiza* based on UPLC-Qqq-MS and antioxidant activities. Molecules.

[7] Zhao Q., Song Z., Fang X., Pan Y., Guo L., Liu T., Wang J. (2016). Effect of genotype and environment on *Salvia miltiorrhiza* roots using LC/MS-based metabolomics. Molecules.

[8] He C.E., Lu L.L., Jin Y., Wei J.H., Christie P. (2013). Effects of nitrogen on root development and contents of bioactive compounds in *Salvia miltiorrhiza* Bunge. Crop Sci..

[9] Zhao Y.J., Chen S.B., Gao G.Y., Feng Y.X., Yang S.L., Xu L.Z., Du L.J., Lin J., Li M. (2004). Content of inorganic elements of *Salvia miltiorrhiza* root in different area and physicochemical properties of its growing soil. Zhongguo Zhong Yao Za Zhi.

[10] Huynh N.T., Van Camp J., Smagghe G., Raes K. (2014). Improved release and metabolism of flavonoids by steered fermentation processes: A review. Int. J. Mol. Sci..

[11] Dey T.B., Chakraborty S., Jain K.K., Sharma A., Kuhad R.C. (2016). Antioxidant phenolics and their microbial production by submerged and solid state fermentation process: A review. Trends Food Sci. Technol..

[12] Handayani R., Sulistyo J., Rahayu R.D. (2009). Extraction of coconut oil (*Cocos nucifera* L.) through fermentation system. Biodiversitas.

[13] Lee M., Cho J.Y., Lee Y.G., Lee H.J., Lim S.I., Park S.L., Moon J.H. (2015). Bioconversion of Capsaicin by *Aspergillus oryzae*. J. Agric. Food Chem..

[14] Razak D.L.A., Rashid N.Y.A., Jamaluddin A., Sharifudin S.A., Kahar A.A., Long K. (2017). Cosmeceutical potentials and bioactive compounds of rice bran fermented with single and mix culture of *Aspergillus oryzae* and *Rhizopus oryzae*. J. Saudi Soc. Agric. Sci..

[15] Lee S., Seo M.H., Oh D.K., Lee C.H. (2014). Targeted metabolomics for *Aspergillus oryzae*-mediated biotransformation of soybean isoflavones, showing variations in primary metabolites. Biosci. Biotechnol. Biochem..

[16] Leyva A., Quintana A., Sánchez M., Rodríguez E.N., Cremata J., Sánchez J.C. (2008). Rapid and sensitive anthrone-sulfuric acid assay in microplate format to quantify carbohydrate in biopharmaceutical products: Method development and validation. Biologicals.

[17] Zhang Q., Zhang J., Shen J., Silva A., Dennis D.A., Barrow C.J. (2006). A simple 96-well microplate method for estimation of total polyphenol content in seaweeds. J. Appl. Phycol..

[18] Chang C.C., Yang M.H., Wen H.M., Chern J.C. (2002). Estimation of total flavonoid content in propolis by two complementary colorimetric methods. J. Food Drug Anal..

[19] Kim M.J., John K.M., Choi J.N., Lee S., Kim A.J., Kim Y.M., Lee C.H. (2013). Changes in secondary metabolites of green tea during fermentation by *Aspergillus oryzae* and its effect on antioxidant potential. Food Res. Int..

[20] Benzie I.F., Strain J.J. (1996). The ferric reducing ability of plasma (FRAP) as a measure of ‘‘antioxidant power’’: The FRAP assay. Anal. Biochem..

[21] Xiang H., Cao F., Ming D., Zheng Y., Dong X., Zhong X., Mu D., Li B., Zhong L., Cao J. (2017). Aloe-emodin inhibits *Staphylococcus aureus* biofilms and extracellular protein production at the initial adhesion stage of biofilm development. Appl. Microbiol. Biotechnol..

[22] Kozikowski B.A., Burt T.M., Tirey D.A., Williams L.E., Kuzmak B.R., Stanton D.T., Morand K.L., Nelson S.L. (2003). The effect of freeze/thaw cycles on the stability of compounds in DMSO. J. Biomol. Screen..

[23] Zeng H., Su S., Xiang X., Sha X., Zhu Z., Wang Y., Guo S., Yan H., Qian D., Duan J. (2017). Comparative analysis of the major chemical constituents in *Salvia miltiorrhiza* roots, stems, leaves and flowers during different growth periods by UPLC-TQ-MS/MS and HPLC-ELSD methods. Molecules.

[24] Thaipong K., Boonprakob U., Crosby K., Cisneros-Zevallos L., Byrne D.H. (2006). Comparison of ABTS, DPPH, FRAP, and ORAC assays for estimating antioxidant activity from guava fruit extracts. J. Food Compos. Anal..

[25] Sung W.S., Jung H.J., Lee I.S., Kim H.S., Lee D.G. (2006). Antimicrobial effect of furaneol against human pathogenic bacteria and fungi. J. Microbiol. Biotechnol..

[26] Mujeeb F., Bajpai P., Pathak N. (2014). Phytochemical evaluation, antimicrobial activity, and determination of bioactive components from leaves of *Aegle marmelos*. BioMed Res. Int..

[27] Kim M.G., Lee H.S. (2014). 1, 2-benzendiol isolated from persimmon roots and its structural analogues show antimicrobial activities against food-borne bacteria. J. Korean Soc. Appl. Biol. Chem..

[28] Scopel M., Abraham W.R., Antunes A.L., Terezinha Henriques A., Macedo J., Jose A. (2014). Mevalonolactone: An inhibitor of *Staphylococcus epidermidis* adherence and biofilm formation. Med. Chem..

[29] Krishnan K.R., James F., Mohan A. (2016). Isolation and characterization of n-hexadecanoic acid from *Canthium parviflorum* leaves. J. Chem. Pharm. Res..

[30] Musa A.M., Ibrahim M.A., Aliyu A.B., Abdullahi M.S., Tajuddeen N., Ibrahim H., Oyewale A.O. (2015). Chemical composition and antimicrobial activity of hexane leaf extract of *Anisopus mannii* (Asclepiadaceae). J. Intercult. Ethnopharmacol..

[31] Huang C.B., George B., Ebersole J.L. (2010). Antimicrobial activity of n-6, n-7 and n-9 fatty acids and their esters for oral microorganisms. Arch. Oral Biol..

[32] Zheng C.J., Yoo J.S., Lee T.G., Cho H.Y., Kim Y.H., Kim W.G. (2005). Fatty acid synthesis is a target for antibacterial activity of unsaturated fatty acids. FEBS Lett..

[33] Matsushita Y.I., Hwang Y.H., Sugamoto K., Matsui T. (2006). Antimicrobial activity of heartwood components of sugi (*Cryptomeria japonica*) against several fungi and bacteria. J. Wood Sci..

[34] Deyab M.A., Abou-Dobara M.I. (2013). Antibacterial activity of some marine algal extracts against most nosocomial bacterial infections. Egypt. J. Exp. Biol. (Bot.).

[35] Kim M.H., Kim S.I., Seo D.W., Ryu J.C., Choi H.Y. (2010). Antioxidant activity of *Salvia miltiorrhiza* Bunge, a novel foodstuff. Mol. Cell Toxicol..

[36] Wen Y.L., Yan L.P., Chen C.S. (2013). Effects of fermentation treatment on antioxidant and antimicrobial activities of four common Chinese herbal medicinal residues by *Aspergillus oryzae*. J. Food Drug Anal..

[37] Xing Y., Cai L., Yin T.P., Chen Y., Yu J., Wang Y.R., Ding Z.T. (2016). Improving the antioxidant activity and enriching salvianolic acids by the fermentation of *Salvia miltiorrhizae* with *Geomyces luteus*. J. Zhejiang Univ. Sci. B.

[38] Dos Santos Oliveira M., Feddern V., Kupski L., Cipolatti E.P., Badiale-Furlong E., de Souza-Soares L.A. (2011). Changes in lipid, fatty acids and phospholipids composition of whole rice bran after solid-state fungal fermentation. Bioresour. Technol..

[39] Li X., Ma M., Xin X., Tang Y., Zhao G., Xiao X. (2019). Efficient acylation of gastrodin by *Aspergillus oryzae* whole-cells in non-aqueous media. RSC Adv..

[40] Khmelnitsky Y.L., Budde C., Arnold J.M., Usyatinsky A., Clark D.S., Dordick J.S. (1997). Synthesis of water-soluble paclitaxel derivatives by enzymatic acylation. J. Am. Chem. Soc..

[41] Guo Q.Q., Zeng J.H., Lu Y., Shu X.G. (2013). Effects of solubility, thermal stability and antioxidant properties of acylating dihydromyricetin. Advanced Materials Research.

[42] Li R., Narita R., Ouda R., Kimura C., Nishimura H., Yatagai M., Fujita T., Watanabe T. (2018). Structure-dependent antiviral activity of catechol derivatives in pyroligneous acid against the encephalomycarditis virus. RSC Adv..

[43] Ralston A.W., Hoerr C.W. (1942). The solubilities of the normal saturated fatty acids. J. Org. Chem..

[44] Sedgwick R.S., Hoerr C.W., Harwood H.J. (1952). Solubilities of saturated fatty acid esters. J. Org. Chem..

