# Supplementary material for: Enhancement of Antioxidant and Antibacterial Activities of Salvia miltiorrhiza Roots Fermented with Aspergillus oryzae
Source: Foods. 2020 Jan 1;9(1):34. doi: 10.3390/foods9010034 (PMC7023044; doi:10.3390/foods9010034)
Supplement: Supplementary file 1 [file foods-09-00034-s001.pdf]

Supplementary data

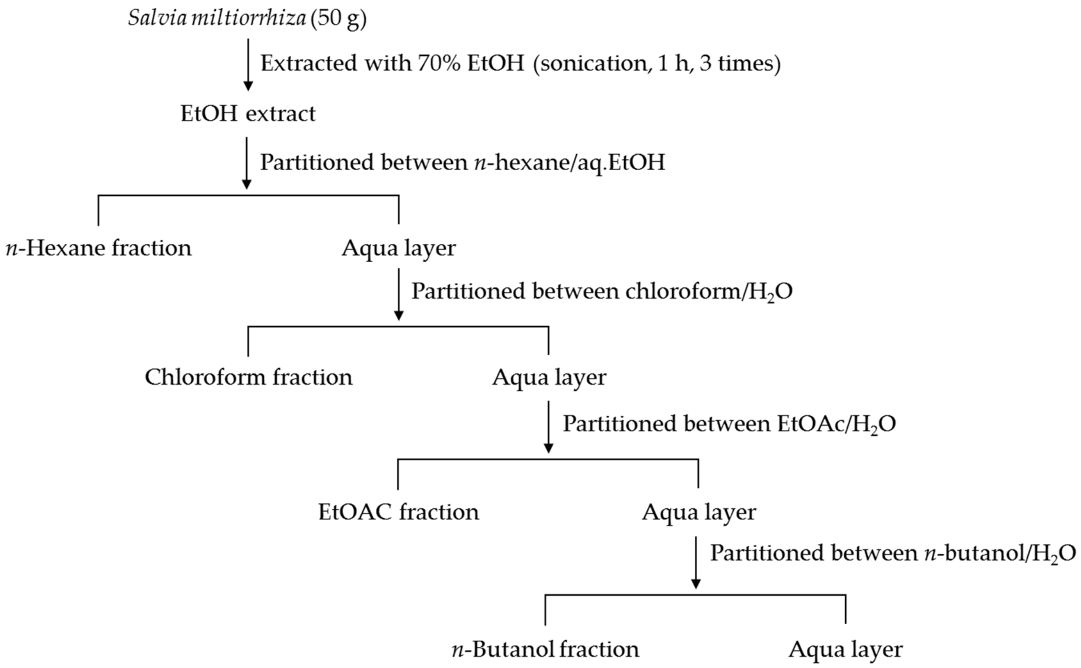

Supplementary Figure 1. Sequential fractionation of EtOH extract with organic solvents.

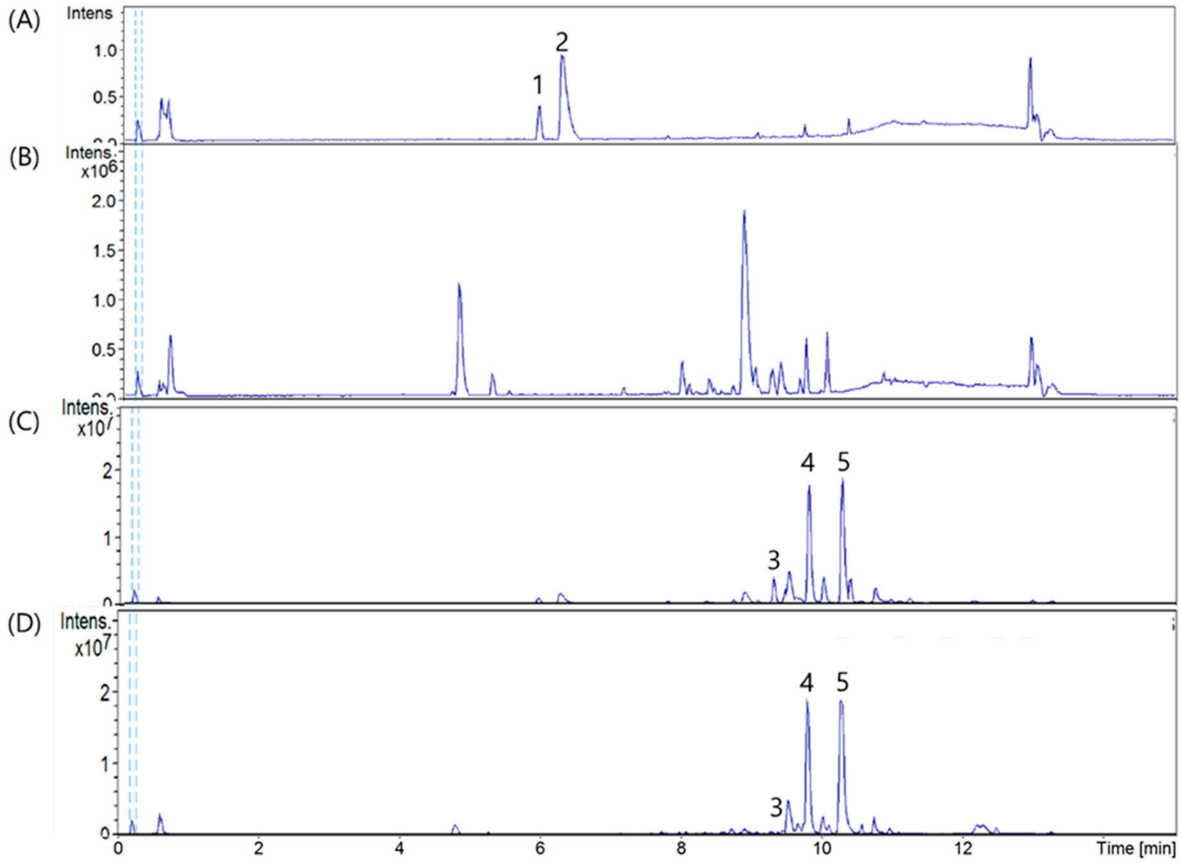

Supplementary Figure 2. LC-MS chromatogram of SME (A and C) and SMBE (B and D). (A and B) SME and SMBE in positive ESI mode; (C and D) SME and SMBE in negative ESI mode (1, rosmarinic acid; 2, salvianolic acid B; 3, dihydrotanshinone I; 4, cryptotanshinone; 5, tanshinone IIA).

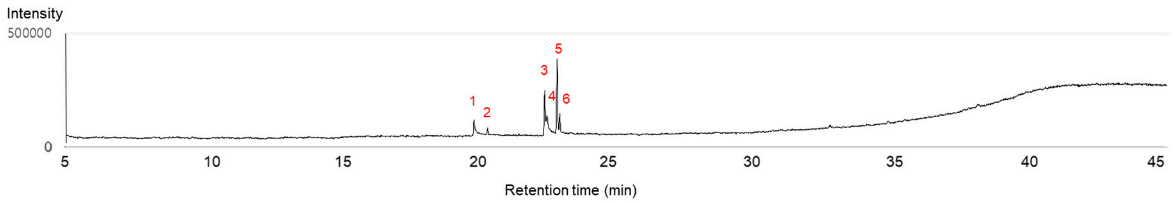

Supplementary Figure 3. GC-MS chromatogram of AoryE (1, hexadecanoic acid; 2, hexadecanoic acid, ethyl ester; 3, (9E,12E)-methyl octadeca-9,12-dienoate; 4, octadec-9-enoic acid; 5, (9Z,12Z)-ethyl octadeca-9,12-dienoate; 6, 9-octadecenoic acid (Z)-, ethyl ester).

Supplementary Table 1. Compounds detected by GC-MS analysis of AoryE

Peak No.	RT (min)	Area	Name of compound
1	19.8	240,808	hexadecanoic acid
2	20.3	68,819	hexadecanoic acid, ethyl ester
3	22.4	610,406	(9E,12E)-methyl octadeca-9,12-dienoate
4	22.5	286,373	octadec-9-enoic acid
5	22.8	818,101	(9Z,12Z)-ethyl octadeca-9,12-dienoate
6	22.9	233,350	9-octadecenoic acid (Z)-, ethyl ester
